# Functional data analysis-based yield modeling in year-round crop cultivation

**DOI:** 10.1093/hr/uhae144

**Published:** 2024-05-24

**Authors:** Hidetoshi Matsui, Keiichi Mochida

**Affiliations:** Faculty of Data Science, Shiga University, Banba, Hikone, Shiga 522-8522, Japan; RIKEN Center for Sustainable Resource Science, Yokohama 230-0045, Japan; Kihara Institute for Biological Research, Yokohama City University, Yokohama 244-0813, Japan; School of Information and Data Sciences, Nagasaki University, Nagasaki 852-8521 Japan

## Abstract

Crop yield prediction is essential for effective agricultural management. We introduce a methodology for modeling the relationship between environmental parameters and crop yield in longitudinal crop cultivation, exemplified by strawberry and tomato production based on year-round cultivation. Employing functional data analysis (FDA), we developed a model to assess the impact of these factors on crop yield, particularly in the face of environmental fluctuation. Specifically, we demonstrated that a varying-coefficient functional regression model (VCFRM) is utilized to analyze time-series data, enabling to visualize seasonal shifts and the dynamic interplay between environmental conditions such as solar radiation and temperature and crop yield. The interpretability of our FDA-based model yields insights for optimizing growth parameters, thereby augmenting resource efficiency and sustainability. Our results demonstrate the feasibility of VCFRM-based yield modeling, offering strategies for stable, efficient crop production, pivotal in addressing the challenges of climate adaptability in plant factory-based horticulture.

## Introduction

Crop yield prediction is one of the long-standing challenges in modern agriculture. An accurate method to predict crop yield facilitates making management decisions to improve the cost–benefit balance in agriculture, aiming to make it more profitable and sustainable to cope with the emergence of climate change and world population growth [1, 2]. In recently developed information technology-assisted frameworks for factory style precision agriculture, computational modeling to predict crop growth and forecast crop yield plays a pivotal role in preemptive agricultural management [3]. However, modeling crop growth is challenging especially for year-round crop cultivation because crop growth depends on interactions of various factors including crop genotype as well as seasonally fluctuating environmental conditions and management practices [4].

To predict crop yield based on various factors that may influence crop growth, many modeling approaches have been proposed. The recent emergence and popularization of artificial intelligence-related techniques have facilitated predictive modeling even in agricultural sectors [5]. Indeed, machine learning-based modeling has enabled us to rapidly develop a model to predict agricultural outcomes based on various factors. However, such machine learning-based approaches often provide less interpretable models due to their complexity and black-box nature [6]. In contrast, statistical modeling-based approaches often facilitate a better understanding, with insights into relationships between outcomes and explanatory factors [7]. Several statistical models have been considered and implemented for representing the relationship between crop yield and environmental factors [8], and their effectiveness has been demonstrated [9]. Methods for predicting crop yield of year-round cultivation such as strawberries and tomatoes have been developed as improvements of data acquisition technology. Saito *et al*. [10] developed a growth model of greenhouse tomatoes using data for environmental factors and leaf areas, based on botanical knowledge. Kim *et al*. [11] approached the prediction of the yield of strawberries using mixed effect models. Chen *et al*. [12] and [13] independently developed the method for predicting the yield of strawberries by detecting flowers using deep neural networks.

Functional data analysis (FDA) provides a statistical framework that allows us to deal with each individual of data as a continuum (curve or surface) represented by a function transformed from a series of continuously recorded data [14, 15]. For example, when daily temperatures are observed over a year for many years, a curve representing the time-course pattern of the temperature in each year is considered as a series of data. The essential idea behind FDA is to transform data from repeated measurements over time into a function for individuals and then analyze a set of functions. Recently, FDA has been widely applied to analyze time-series data to represent complex time-dependent phenomena in various fields, including public health [16], life science [17], and socioeconomics [18]. Other applications of FDA to several fields are summarized in Ullah and Finch [19]. Because crop growth and development can also be explained as a function of time during the growth period, it has naturally led us to consider FDA-based representation of environmental parameters to predict crop yield, based on various time-dependent factors that affect crop growth and yield [20].

In this study, we address the intricacies of yield modeling of strawberries and tomatoes cultivated in a natural-light plant factory. While this cultivation strategy unlocked the potential for cost-effective, continuous production and enhanced productivity, it also introduces a challenge in the management of cultivation throughout the year. Since the growth and yield of strawberries and tomatoes are subject to daily and seasonal environmental changes, illustrating longitudinal environmental impact, the development of a yield prediction method based on these dynamics is essential. To dissect and understand the impact of the environment on crop yields in year-round cultivation, we collected data on the daily yield of strawberries and tomatoes and ambient environmental parameters within natural-light horticulture facilities. Employing FDA with our rich dataset, we quantitatively examined the relationship between the environmental factors and crop yields and crafted a predictive model that forecasts the yield based on such environmental influences, offering insights into the environmental impacts on crop growth over different seasons. Advantages of our method are that it provides interpretable relationships between environmental factors and crop yields and that it is available for any kind of data for year-round cultivation. Our FDA-based approach, while focusing on the interplay between environmental factors and crop yield, sets a precedent for broader applications in various scenarios in agriculture, where time-series datasets are acquirable, thereby contributing to the advancement of sustainable practices in crop production.

## Results and discussion

### Strawberry dataset

**Figure 1:**
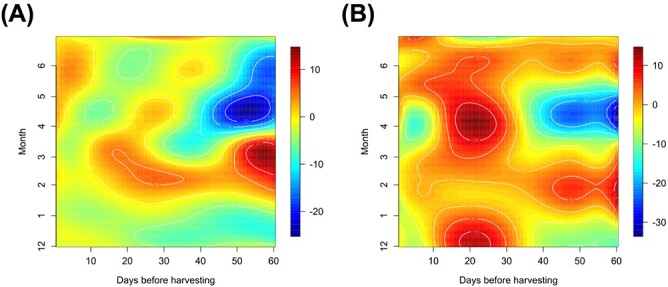
Estimated coefficient surfaces for temperature and solar radiation for strawberry yield data. The contour maps illustrate the changing influence of temperature (A) and solar radiation (B) on the strawberry yield at harvest dates (vertical axis) during the 60 days cultivation period before harvesting (horizontal axis).

With the selected model, we examined the estimated coefficient surfaces ${\hat{\beta}}_j\left(s,t\right)\ \left(j=1,2\right)$ for temperature and solar radiation (Fig. 1). These surfaces provide insights into the temporal distribution of environmental impact on strawberry yield. Assessing the temperature’s coefficient surface, we observed that positive peaks in the coefficient surface approximately from 30 to 60 days before the harvest date around March, which indicates that higher temperatures around this period positively influence the yield (Fig. 1a). Conversely, negative peaks approximately 50–60 days before the harvest date from April to June suggest that higher temperatures at this stage negatively impact the yield. These findings suggest that both higher and lower temperatures, at specific times in the growth cycle, may have beneficial effects on strawberry productivity. For solar radiation, from 1 to 30 days before the harvest tends to have a positive influence on the harvest for almost all season, whereas that from 40 to 60 days before cultivation tends to have a negative impact around April (Fig. 1B). These results indicate that higher temperature around January and February (from 30 to 60 days before harvest date in March) and lower temperature around February (from 50 to 60 days before harvest date in April) could be associated with increased strawberry yields, while higher solar radiation for 30 days before harvest and lower solar radiation around February (from 40 to 60 days before harvest date in April) may also boost its yields. These insights quantify the dynamic contributions of multiple environmental factors such as temperature and solar radiation to yield at various stages of the growth cycle.

### Tomato dataset

**Figure 2:**
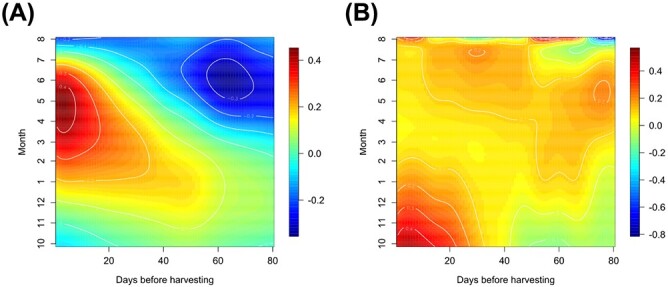
Estimated coefficient surfaces for temperature and solar radiation for tomato yield data. The contour maps illustrate the changing influence of temperature (A) and solar radiation (B) on the tomato yield at harvest dates (vertical axis) during the 80 days cultivation period before harvesting (horizontal axis).

We also examined the estimated coefficient surfaces ${\hat{\beta}}_j\left(s,t\right)\ \left(j=1,2\right)$ for temperature and solar radiation (Fig. 2). A positive peak was observed in the temperature coefficient surface approximately 1–20 days before the harvest from January to July, suggesting that higher temperatures during these periods positively influence yield (Fig. 2A). Conversely, a negative peak was detected from 50 to 80 days before the harvest from May to July, suggesting that lower temperatures at this stage also positively contribute to the tomato yield. In the solar radiation coefficient surface, we found that increased solar radiation, particularly from one to 30 days before harvest around October to January, positively influences tomato yields (Fig. 2B). Our findings highlight two key aspects: (i) tomato plants exhibit temporal interactions with ambient temperature and solar radiation and (ii) the effect of this interplay between tomatoes and temperature on yield varies throughout the cultivation season.

**Figure 3:**
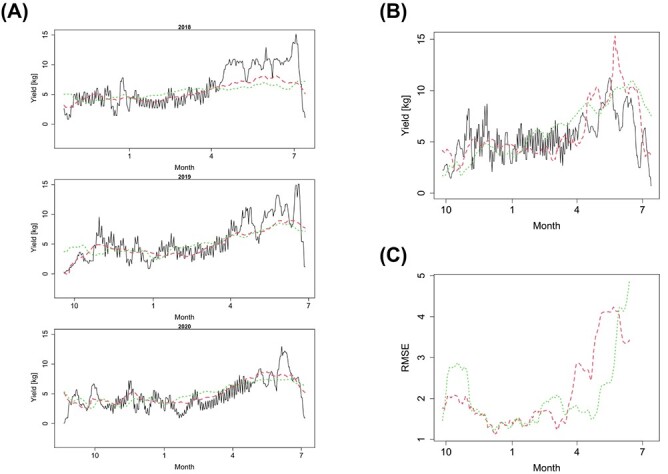
Predictions of tomato yield. The line graphs show the results of yield predictions for the first 3 years (A) and the fourth year (B), where the solid curves represent the actual measured yield values, while the dashed and dotted curves indicate the predicted values from VCFRM and FRM, respectively. (A) The left graph displays the relationship between actual and predicted tomato yields over three years of datasets as training data. (B) The top right graph compares the actual and predicted yields for a future dataset using VCFRM and FRM. (C) RMSE of 30 days for the yield prediction by VCFRM (dashed curve) and FRM (dotted curve).

Figure 3 shows the comparison among observed tomato yields and predicted yields by VCFRM and FRM for the training and test datasets. Note that the predicted yields for the training datasets in Fig. 3A are obtained using the data for the first 3 years, whereas Fig. 3B shows the predicted yields for the test data. Figure 3C depicts trajectories of the root mean squared errors (RMSEs) of the prediction results for VCFRM and FRM in Fig. 3B, averaged by those for 30 days. The RMSE for the $i$th day is defined by

$$RMS{E}_i=\sqrt{\frac{1}{30}{\sum}_{j=i}^{i+30}{\left({y}_j\left({t}_j\right)-{\hat{y}}_j\left({t}_j\right)\right)}^2},$$

where ${\hat{y}}_i\left({t}_i\right)$ is the predicted yield by VCFRM or FRM. The results for the VCFRM exhibit good accuracy in yield prediction from autumn to winter, while it encounters challenges in accurately forecasting yields from spring to summer (Fig. 3C). In the dataset from the fourth year, which are predicted as the test data, the actual tomato yields in spring and summer were notably lower compared to the preceding three years, even though the temperature and solar radiation during the fourth year did not differ significantly from other years (Fig. 6). In fact, the predicted yields in spring and summer, as estimated by our VCFRM-based model, aligned closely to the actual yields in spring and summer in the three years rather than those in the fourth year. In contrast, the FRM-based model does not capture this trend. This may be attributed to the VCFRM’s ability to capture the specific relationship between environmental factors and crop yields during the spring and summer seasons, unlike the FRM-based model. Despite the strong correlation between temperature and solar radiation with yield during these seasons, as depicted in Fig. 6, our model’s performance implies the potential influence of other critical factors that might significantly impact tomato yields over these months.

**Figure 4:**
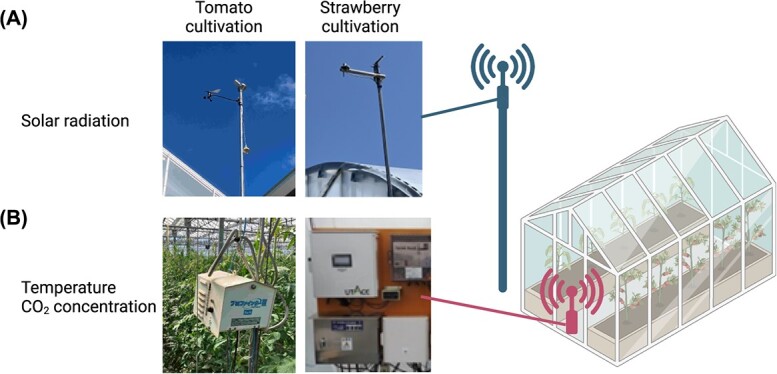
Environmental monitoring system in strawberry and high-wire tomato cultivation greenhouse. (A) External sensor unit continuously monitors environmental parameters including solar radiation and wind speed. (B) Internal sensor unit placed within the greenhouse tracks microclimate parameters including temperature and CO_2_ concentration. These figures were created with BioRender.com.

**Figure 5:**
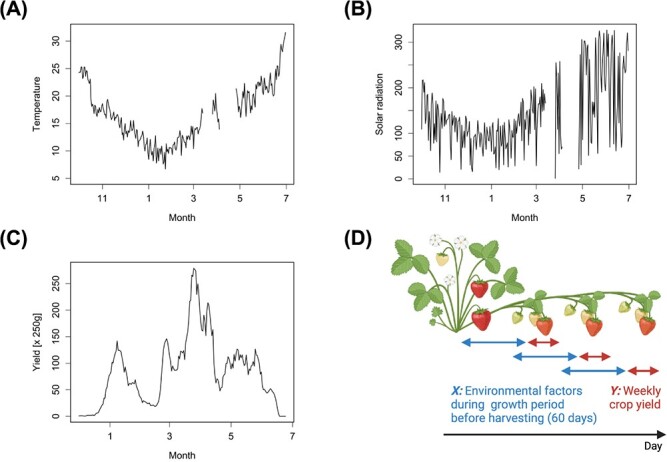
Datasets of yield and environmental parameters for strawberry cultivation. (A) Temporal fluctuations in temperature within the greenhouse. (B) Solar radiation data recorded over the same period as (A), illustrating the intensity of sunlight. (C) Weekly strawberry yields during the harvest periods. (D) A schematic diagram illustrating the relationship between environmental factors during the growth period (X: predictors, up to 60 days before harvesting) as seen in (A) and (B), and strawberry fruit development cycle, leading to the weekly crop yield (Y: response) as seen in (C). Each individual observation corresponding to X and Y is observed for each date of strawberry harvest. (D) was created with BioRender.com.

**Figure 6:**
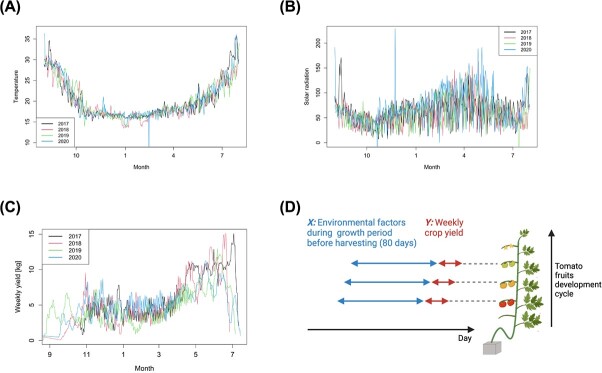
Datasets of yield and environmental parameters for tomato cultivation. (A) Temporal fluctuations in temperature within the greenhouse for four years. (B) Solar radiation data recorded over the same period as (A), illustrating the intensity of sunlight. (C) Weekly tomato yields during the harvest periods. (D) A schematic diagram illustrating the relationship between environmental factors during the growth period (*X*: predictors, up to 80 days before harvesting) as seen in (A) and (B), and tomato fruit development cycle, leading to the weekly crop yield (*Y*: response) as seen in (C). Each individual observation corresponding to X and Y is observed for each date of tomato harvest. (D) was created with BioRender.com.

In this study, with the yield datasets for strawberries and tomatoes and the ambient environmental datasets from a commercial greenhouse-type horticulture facility, we demonstrated the feasibility of an FDA-based modeling approach to represent the relation between yields of strawberries and tomatoes and the environment. In Internet-of-Things-assisted smart agriculture, various types of time-series datasets related to crop growth and ambient environments are simultaneously collected in greenhouses as well as arable fields [23–25]. FDA allowed us to examine the time-dependent impact of various factors on crops, as demonstrated in this study, providing insights into the temporal interactions between crops and their environments (Figs 1 and 2). Under greenhouse and arable field conditions, crops are continuously and simultaneously exposed to environmental fluctuations throughout their life cycle [26, 27]. The high interpretability of FDA-based modeling facilitates identifying critical time points in crop growth and environmental responses, providing new insights into crop–environment interactions. These insights are essential for forecasting agronomic outcomes that emerge in later stages of crops. Our method is applicable across a range of agricultural scenarios where time-series data are available for exploring the relationship between crop yield and temporal environmental factors. Our FDA-based analysis provides crucial insights into temporal crop-environment interactions, pinpointing varieties that are more resilient or sensitive to seasonal environmental changes. These insights enable us to strategically select and cultivate crop varieties better suited to diverse environmental conditions, which aids in maintaining overall yield and profitability even under challenging environmental circumstances. Therefore, this methodology presents practical solutions that facilitate sustainable and efficient crop production in an ever-changing climate.

## Materials and methods

### Datasets of yield and environmental parameters of strawberries

We analyzed dataset for strawberries (*Fragaria x magna* Thuill cv. Akihime) grown in a greenhouse-type horticulture facility at Shiga, Japan (35°111′N, 135°997′E). Strawberry plants were grown with long-term cultivation and harvested almost every day from December to June. The daily yields of strawberries are measured in the units of packages (250 g per package). We used a dataset obtained for 212 days from 2021 to 2022. The dataset also contains the greenhouse environment such as temperature and solar radiation. Temperature and solar radiation were measured every 2 minutes from inside the greenhouse (Fig. 4).

### Datasets of yield and environmental parameters of tomato

We also analyzed a yield dataset for tomatoes (*Lycopersicon esculentum* Mill. cv. House Momotaro) grown in a commercial greenhouse-type horticulture facility at Hyogo, Japan (34°863′N, 135°235′E). Tomato plants were grown with high wire-style year-round cultivation and harvested almost every day except the farm's holidays from October to July annually. The data contain daily yields of tomatoes at a unit area (kg). We used these datasets for 4 years, which in total accounts for 1172 days.

We also conducted monitoring of the greenhouse environment, collecting data on temperature, solar radiation, and the degree of closure of the greenhouse shading curtains. Temperature and solar radiation were measured every minute from inside and outside, respectively (Fig. 4), which amounts to about 2.1 million data points in total. We also continuously tracked the degree of closure of the shading curtains. The data of the degree of the shading curtains’ closure serves as a surrogate for the solar radiation for tomato plants within the greenhouse, as its closure restricts the incoming sunlight.

### Functional data analysis

To continuously quantify the dynamic contribution of environmental factors until the harvest date by a regression model, we used the daily crop yield as a response variable and the environmental factors (temperature and solar radiation) for 60 days in strawberry and 80 days in tomato prior to the harvest date as predictor variables (Figs 5 and 6). To smooth the daily yield datasets and avoid the effect of missing data due to facility holidays, we used the moving average of the yield up to seven days from the harvest date, while to smooth the environmental datasets, we used the average values for each hour for each of the environmental datasets. Using basis expansion techniques, we then transformed these data into smooth functions [15]. Details of transforming the environmental data into functions based on the basis expansion are given in the supplemental materials (Supplementary data S1).

Then, we represented the relationship with the yields using a varying-coefficient functional regression model (VCFRM; [21]). Suppose that ${y}_i\left({t}_i\right)$ is the moving average of the crop yield up to seven days from the observed day ${t}_i$ with observation index $i=1,\dots, n$ and ${x}_{ij}(t)$ is the $j\mathrm{th}$ environmental factor transformed into functional data for the $i\mathrm{th}$ observation. The correspondence of ${x}_{ij}(t)$ and ${y}_i\left({t}_i\right)$ is shown in Figs 5(D) and 6(D). We also centered both ${x}_{ij}(t)$ and ${y}_i\left({t}_i\right)$ so that ${\sum}_{i=1}^n{x}_{ij}(t)/n=0$ for all $j$ and${\sum}_{i=1}^n{y}_i\left({t}_i\right)/n=0.$ Then, the following VCFRM is used as a statistical model that represents the relationship between environmental factors and crop yield:

$$ {y}_i\left({t}_i\right)={\sum}_{\mathrm{j}=1}^2\int{x}_{ij}(t){\beta}_j\left(s,{t}_i\right) ds+{\varepsilon}_i, $$

where ${\beta}_j\left(s,{t}_i\right)$ is a coefficient function for the $j\mathrm{th}$ environmental factor. These are the unknown parameters of the model. Furthermore, ${\varepsilon}_i$ is an error, where $\boldsymbol{\varepsilon} ={\left({\varepsilon}_1,\dots, {\varepsilon}_n\right)}^T$ has mean zero vector and variance–covariance matrix $\varSigma$. In applying the VCFRM, we operate under key assumptions: Firstly, the impact of environmental factors on yield varies smoothly over time during the days preceding a given harvest date—80 days in tomatoes and 60 days in strawberries in this study. Secondly, this environmental impact also varies smoothly with the season. Lastly, we assume that the environmental impact is linear at fixed time points. Together, these assumptions enable the exploration of the temporal effects of multiple environmental parameters across a diverse range of horticultural crops engaged in long-term such as year-round cultivation.

We estimated the unknown coefficients ${\beta}_j\left(s,{t}\right)$ by the regularization method with smoothness penalties and then evaluated the estimation model. Details of the method for estimating the model are given in the supplemental materials (Supplementary data S2). Through an information criterion, we selected the most appropriate tuning parameters that control the smoothness of coefficient surfaces ${\beta}_j\left(s,t\right)$. Details of the tuning parameter selection by the information criterion are given in the supplemental materials (Supplementary data S3). In our method, we expanded the established VCFRM by incorporating multiple predictors to integrate various environmental factors. Additionally, we estimated the model using a regularization method that imposes smoothness in both the $s$ and $t$ dimensions for ${\beta}_j\left(s,t\right)$, allowing the effect of the environmental factors on yield to exhibit a smooth variation over time toward cultivation periods and across seasons. Then, we evaluated the estimated model using the information criterion derived from the prediction viewpoint [22].

We used the respective datasets for strawberries and tomatoes to estimate and analyze regression coefficients functions ${\beta}_j\left(s,t\right)$ for each of the crops. Additionally, for the tomato dataset specifically, to assess the predictive performance of our statistical model, we conducted an experiment to predict future yields using its past data in line with the operation of the tomato horticulture facility. First, we treated the data with the observation indices $i=1,\dots, {n}_0-6$ as the training data for an integer ${n}_0$, whereas we used the single observation $i={n}_0$ as the test data so that the farmer can predict the total yield for the next one week using the data currently available. Note that ${y}_i\left({t}_i\right)$ is the average of the yield for 7 days from the day ${t}_i$ and that we used the observations $i=1,\dots, {n}_0-6$ as the training data rather than $i=1,\dots, {n}_0-1$ to prevent data leak. We repeated the analysis by assigning the observation indices of the fourth year to ${n}_0$, that is, we treated the data for the first 3 years and a part of the fourth year as the training data. We used R 4.3.2 to implement the statistical analysis and used R package “fda” for the transformation of the observed data into functional data.

Moreover, we compared our prediction results for the tomato dataset with those of the functional regression model (FRM, [15]), which is given by


$$ {y}_i\left({t}_i\right)={\sum}_{\mathrm{j}=1}^2\int{x}_{ij}(s){\beta}_j(s) ds+{\varepsilon}_i. $$

This model also expresses the relationship between time-course environmental factors and crop yield. However, unlike the VCFRM, it does not account for seasonal variation in the relationship.

## Supplementary Material

Web_Material_uhae144

## Data Availability

The data underlying this article cannot be shared publicly due to the trade secret.

## References

[1] Iaksch J , Fernandes E, Borsato M. Digitalization and big data in smart farming – a review. J Manag Anal. 2021;8:333–49

[2] Telagam N , Kandasamy N, Arun Kumar M. Review on smart farming and smart agriculture for society: post-pandemic era. In: Chakraborty C, ed. Green technological innovation for sustainable smart societies. Springer: Cham, 2021,

[3] Hao S , Ryu D, Western A. et al. Performance of a wheat yield prediction model and factors influencing the performance: a review and meta-analysis. Agric Syst. 2021;194:103278

[4] Ray D , Gerber J, MacDonald G. et al. Climate variation explains a third of global crop yield variability. Nat Commun. 2015;6:5989 25609225 10.1038/ncomms6989 PMC4354156

[5] van Klompenburg T , Kassahun A, Catal C. Crop yield prediction using machine learning: a systematic literature review. Comput Electron Agric. 2020;177:105709

[6] Liakos KG , Busato P, Moshou D. et al. Machine learning in agriculture: a review. Sensors. 2018;18:2674 30110960 10.3390/s18082674 PMC6111295

[7] Kang Y , Ozdogan M, Zhu X. et al. Comparative assessment of environmental variables and machine learning algorithms for maize yield prediction in the US Midwest. Environ Res Lett. 2020;15:064005

[8] Kang Y , Khan S, Ma X. Climate change impacts on crop yield, crop water productivity and food security – a review. Prog Nat Sci. 2009;19:1665–74

[9] Lobell DB , Burke MB. On the use of statistical models to predict crop yield responses to climate change. Agric For Meteorol. 2010;150:1443–52

[10] Saito T , Kawasaki Y, Ahn D-H. et al. Prediction and improvement of yield and dry matter production based on modeling and non-destructive measurement in year-round greenhouse tomatoes. Hort J. 2020;89:425–31

[11] Kim S , Jo JS, Luk V. et al. Estimating the impact of environmental management on strawberry yield using publicly available agricultural data in south Korea. PeerJ. 2023, 2023;11:e15390 37193021 10.7717/peerj.15390 PMC10183158

[12] Chen Y , Lee WS, Gan H. et al. Strawberry yield prediction based on a deep neural network using high-resolution aerial Orthoimages. Remote Sens. 2019;11:1584

[13] Yoon S , Jo JS, Kim SB. et al. Prediction of strawberry yield based on receptacle detection and Bayesian inference. Heliyon. 2023;9:e14546 36967973 10.1016/j.heliyon.2023.e14546 PMC10036644

[14] Kokoszka P , Reimherr M. Introduction to functional data analysis. Boca Raton: CRC Press; 2017:

[15] Ramsay J , Silverman B. Functional data analysis. 2nd ed. New York: Springer; 2005:

[16] Boschi T , Di Iorio J, Testa L. et al. Functional data analysis characterizes the shapes of the first COVID-19 epidemic wave in Italy. Sci Rep. 2021;11:17054 34462450 10.1038/s41598-021-95866-y PMC8405612

[17] Kayano M , Matsui H, Yamaguchi R. et al. Gene set differential analysis of time course expression profiles via sparse estimation in functional logistic model with application to time-dependent biomarker detection. Biostatistics. 2016;17:235–48 26420796 10.1093/biostatistics/kxv037

[18] Padilla-Segarra A , González-Villacorte M, Amaro IR. et al. Brief review of functional data analysis: a case study on regional demographic and economic data. In: Information and Communication Technologies. Cham: Springer International Publishing, 2020,163–76

[19] Ullah S , Finch CF. Applications of functional data analysis: a systematic review. BMC Med Res Methodol. 2013;13:43 23510439 10.1186/1471-2288-13-43 PMC3626842

[20] Wong RW , Li Y, Zhu Z. Partially linear functional additive models for multivariate functional data. J Am Stat Assoc. 2019;114:406–18

[21] Wu Y , Fan J, Müller HG. Varying-coefficient functional linear regression. Bernoulli. 2010;16:730–58

[22] Konishi S , Kitagawa G. Information criteria and statistical modeling. New York: Springer; 2008:

[23] Quy VK , Hau NV, Anh DV. et al. IoT-enabled smart agriculture: architecture, applications, and challenges. Appl Sci. 2022;12:3396

[24] Rayhana R , Xiao G, Liu Z. Internet of things empowered smart greenhouse farming. IEEE J Radio Freq Identif. 2020;4:195–211

[25] Wolfert S , Ge L, Verdouw C. et al. Big data in smart farming – a review. Agric Syst. 2017;153:69–80

[26] Mochida K , Lipka AE, Hirayama T. Exploration of life-course factors influencing phenotypic outcomes in crops. Plant Cell Physiol. 2020a;61:1381–3 32603418 10.1093/pcp/pcaa087 PMC7434585

[27] Mochida K , Nishii R, Hirayama T. Decoding plant–environment interactions that influence crop agronomic traits. Plant Cell Physiol. 2020b;61:1408–18 32392328 10.1093/pcp/pcaa064 PMC7434589

