# Supplementary material for: Functional data analysis-based yield modeling in year-round crop cultivation
Source: Hortic Res. 2024 May 24;11(7):uhae144. doi: 10.1093/hr/uhae144 (PMC11234900; doi:10.1093/hr/uhae144)
Supplement: Web_Material_uhae144 [file web_material_uhae144.pdf]

Supplements of "Functional data analysis-based yield modeling in year-round crop cultivation"

Hidetoshi Matsui[1] and Keiichi Mochida[2,3,4]

1 Faculty of Data Science, Shiga University
Hikone, Shiga 522-8522, Japan
hmatsui@biwako.shiga-u.ac.jp
2 RIKEN Center for Sustainable Resource Science
Yokohama, Kanagawa 230-0045, Japan
3 Kihara Institute for Biological Research, Yokohama City University
Yokohama 230-0045, Japan
4 School of Information and Data Sciences, Nagasaki University
Nagasaki 852-8521, Japan

1 Transforming observed data into functional data

We describe the method for transforming the observed time-course data for environmental factors such as temperature and solar radiation into functional data. Suppose we have N_{ij} repeatedly measurements $x_{ij1}, \dots, x_{ijN_{ij}}$ at time points $s_{ij1}, \dots, s_{ijN_{ij}}$, respectively, for the i-th observation and the j-th variable. In our analysis, the i and j correspond to the day the crop yield is observed and the environmental factor, respectively, and N_{ij} is the number of days before the i-th day. Our goal here is to express the data $\{(s_{ij\alpha}, x_{ij\alpha});\ \alpha = 1, \dots, N_{ij},\ \ i = 1, \dots, n,\ j = 1, 2, s_{i\alpha} \in \mathcal{S} \subset \mathbb{R}\}$ as a set of smooth functions $\{x_{ij}(s);\ i = 1, \dots, n,\ \ j = 1, 2,\ \ s \in \mathcal{S}\}$ by an appropriate smoothing technique.

Suppose the observation with respect to the i-th observation and the j-th variable, $\{(x_{ij\alpha}, s_{ij\alpha});\ \alpha = 1, \dots, N_i\}$, are drawn from the regression model

$$x_{ij\alpha} = u_{ij}(s_{ij\alpha}) + e_{ij\alpha}, \quad \alpha = 1, \cdots, N_{ij}, \tag{1}$$

where $u_{ij}(s)$ is an unknown smooth function and the errors $e_{ij\alpha}$ are independently, normally distributed with mean zero and variance τ_{ij}^2. We also assume that the function $u_{ij}(s)$ can be expressed by basis expansions:

$$u_{ij}(s) = \sum_{k=1}^{m_j} w_{ijk}\phi_{jk}(s), \tag{2}$$

where $w_{ij1}, \dots, w_{ijm_j}$ are unknown coefficients to be estimated, and $\phi_{j1}(s), \dots, \phi_{jm_j}(s)$ are basis functions. Several basis functions such as Fourier series or B-splines can be used for $\phi_k(s)$ $(k = 1, \dots, m)$. See Green and Silverman (1994) for details. It follows from (1)

that the statistical model based on the regression model via the basis expansion for the i-th observation and the j-th variable is given by

$$f(x_{ij\alpha}|s_{ij\alpha};\boldsymbol{w}_{ij},\tau_{ij}^2)=\frac{1}{\sqrt{2\pi\tau_{ij}^2}}\exp\left[-\frac{\left\{x_{ij\alpha}-\boldsymbol{w}_{ij}^\top\boldsymbol{\phi}(s_{ij\alpha})\right\}^2}{2\tau_{ij}^2}\right],\quad \alpha=1,\cdots,N_{ij}, \tag{3}$$

where $\boldsymbol{w}_{ij}=(w_{ij1},\ \ldots,\ w_{ijm_j})^\top$ and $\boldsymbol{\phi}_j(s)=(\phi_{j1}(s),\ \ldots,\ \phi_{jm_j}(s))^\top$ are vectors of coefficients and basis functions, respectively, and the superscript $\top$ denotes the transpose of the vector or the matrix.

In the functional data analysis context, functional data are obtained using common basis functions for all i. On the other hand, the smoothness of functions differ from each other, and for this reason, applying the maximum likelihood method can be inappropriate to obtain the estimators of $\boldsymbol{w}_{ij}$ and τ_{ij}^2. Instead, we apply the penalized likelihood method that imposes different amounts of smoothness penalties to each subject (Araki et al., 2009). The penalized likelihood method obtains estimators by maximizing the following penalized log-likelihood function.

$$\ell_{\zeta_{ij}}(\boldsymbol{w}_{ij},\tau_{ij}^2)=\sum_{\alpha=1}^{N_{ij}}\log f(x_{ij\alpha}|s_{ij\alpha};\boldsymbol{w}_{ij},\tau_{ij}^2)-\frac{N_{ij}\zeta_{ij}}{2}\boldsymbol{w}_{ij}^\top\Omega_{ij}\boldsymbol{w}_{ij} \tag{4}$$

where $\boldsymbol{x}_{ij}=(x_{ij1},\ldots,x_{ijN_{ij}})^\top$, Φ_{ij} is an $N_{ij}\times m_j$ matrix defined by $\Phi_{ij}=(\boldsymbol{\phi}_j(s_{ij1}),\ldots,\boldsymbol{\phi}_j(s_{ijN_{ij}}))^\top$. Furthermore, $\zeta_{ij}>0$ is a regularization or smoothing parameter, which adjusts the amount of smoothness and also avoids ill-posed problems, and Ω_{ij} is a $m_j\times m_j$ positive semi-definite matrix. There are several form for the matrix Ω_{ij}, here, for all i, we denote $\Omega_{ij}=D_j^{(2)^\top}D_j^{(2)}$ with an $(m_j-2)\times m_j$ matrix $D_j^{(2)}$, given by

$$D_j^{(2)}=\begin{pmatrix}-1 & 2 & 1 & \cdots & 0\\ \vdots & \ddots & \ddots & \ddots & \vdots\\ 0 & \cdots & -1 & 2 & 1\end{pmatrix},$$

that represents the second-order difference operator so that the penalty term is given as

$$\sum_{k=3}^{m_j}(\Delta^2 w_{ijk})^2=\boldsymbol{w}_{ij}^\top D_j^{(2)^\top}D_j^{(2)}\boldsymbol{w}_{ij}, \tag{5}$$

where Δ is a difference operator given by $\Delta w_{ik}=w_{ijk}-w_{ij(k-1)}$. The first term of the right-hand side of (4) is the likelihood function of the probability model (3) whereas the second term is the penalty for the smoothness of the function $x_{ij}(s)$.

By maximizing (4) with respect to $\boldsymbol{w}_{ij}$ and τ_{ij}^2, we have penalized maximum likelihood estimators

$$\hat{\boldsymbol{w}}_{ij}=(\Phi_{ij}^\top\Phi_{ij}+N_{ij}\zeta_{ij}\hat{\tau}_{ij}^2\Omega_{ij})^{-1}\Phi_{ij}^\top\boldsymbol{x}_{ij},\quad \hat{\tau}_{ij}^2=\frac{1}{N_{ij}}(\boldsymbol{x}_{ij}-\Phi_{ij}\boldsymbol{w}_{ij})^\top(\boldsymbol{x}_{ij}-\Phi_{ij}\boldsymbol{w}_{ij}) \tag{6}$$

Then the observed longitudinal data $\{(x_{ij\alpha}, s_{ij\alpha});\ \alpha = 1, \ldots, N_{ij}\}$ are transformed into functional data expressed as

$$x_{ij}(s) \equiv \hat{u}_{ij}(s) = \sum_{k=1}^{m_j} \hat{w}_{ijk}\phi_{jk}(s). \tag{7}$$

The value of the penalized maximum likelihood estimators (6) and the resulting smoothness of functional data (7) strongly depends on the number of basis functions m_j and the value of regularization parameter ζ_{ij}, so the selection of these values is an important issue. To obtain functions with appropriate smoothness for each individual, model selection criteria (Konishi and Kitagawa, 2008) are used. Here we use a cross-validation for selecting m_j and ζ_{ij}.

The observed longitudinal data for the environmental factors $\{(x_{ij\alpha}, s_{ij\alpha});\ s_{ij\alpha} \in \mathcal{S}$, $i = 1, \ldots, n,\ \alpha = 1,\ j = 1, 2,\ \ldots,\ N_{ij}\}$ are smoothed by the method described above, and then we obtain a functional data set $\{x_{ij}(s);\ i = 1, \ldots, n,\ j = 1, 2\}$.

2 Details of the varying-coefficient functional linear model

Suppose we have n sets of observations $\{x_{ij}(s), y_i(t_i), t_i;\ i = 1, \ldots, n,\ j = 1, \ldots, p,\ s \in \mathcal{S} \subset \mathbb{R},\ t \in \mathcal{T} \subset \mathbb{R}\}$, where $x_{i1}(s), \ldots, x_{ip}(s)$ are p functional predictors, $y_i(t_i)$ is a scalar response that depends on another variable t_i. In our analysis, $y_i(t_i)$ corresponds to the average of the crop yield from day t_i to a week after. Furthermore, $x_{ij}(s)$ $(j = 1, 2)$ are temperature and solar radiation respectively that are transformed into functional data, and their periods are 80 days before day t_i. Then we model the relationship between the predictors and a response by the varying-coefficient functional regression model (VCFRM, Cardot and Sarda, 2008; Wu et al., 2010):

$$y_i(t_i) = \sum_{j=1}^{2} \int_{\mathcal{S}_j} x_{ij}(s)\beta_j(s, t_i)ds + \varepsilon_i, \tag{8}$$

where $\beta_j(s, t)$ $(j = 1, 2)$ are coefficient surfaces. Furthermore, ε_i is an normally distributed error, where $\boldsymbol{\varepsilon} = (\varepsilon_1, \ldots, \varepsilon_n)^\top$ has mean zero vector and variance-covariance matrix Σ. The coefficient function $\beta_j(s, t)$ provides us insights about how the functional predictor $x_{ij}(s)$ relates to the response y_i.

The data corresponding to the environmental factors are longitudinally observed and are not functions in nature. Thus we apply the procedure described in the previous section to transform the longitudinal data into functional data before applying the VCFRM. Then

the longitudinal data for the environmental factors are expressed as

$$x_{ij}(s) = \sum_{k=1}^{m_j^{(1)}} w_{ijk}\phi_{jk}(s) = \boldsymbol{w}_{ij}^\top \boldsymbol{\phi}_j(s), \tag{9}$$

where $m_j^{(1)}$ is the number of basis functions for the jth variable, $\boldsymbol{\phi}_j(s) = (\phi_{j1}(s), \ldots, \phi_{jm_j^{(1)}}(s))^\top$ is a vector of basis functions and $\boldsymbol{w}_{ij} = (w_{ij1}, \ldots, w_{ijm_j^{(1)}})^\top$ is a vector of coefficients of basis function $\boldsymbol{\phi}_j(s)$. The $\boldsymbol{w}_{ij}$ are obtained by the penalized likelihood method described in the previous section, and therefore they are supposed to be known here.

To estimate the coefficient functions $\beta_j(s,t)$ in the VCFRM (8), we assume that the $\beta_j(s,t)$ are expressed by basis expansions as follows.

$$\beta_j(s,t) = \sum_{k=1}^{m_j^{(1)}} \sum_{l=1}^{m_j^{(2)}} \phi_{jk}(s) b_{jkl} \psi_{jl}(t) = \boldsymbol{\phi}_j(s)^\top B_j \boldsymbol{\psi}_j(t), \tag{10}$$

where $\boldsymbol{\psi}_j(t) = (\psi_{j1}(t), \ldots, \psi_{jm_j^{(2)}}(t))^\top$ is a vector of basis functions, $m_j^{(2)}$ is a number of basis functions $\boldsymbol{\psi}_j(t)$, and $B_j = (b_{jkl})_{kl}$ is a matrix of unknown parameters.

By applying the assumptions of basis expansions (9) and (10), the VCFRM is expressed by

$$\begin{aligned} y_i &= \sum_{j=1}^{2} \boldsymbol{w}_{ij}^\top \Phi_j B_j \boldsymbol{\psi}_j(t_i) + \varepsilon_i \\ &= \sum_{j=1}^{2} \left\{ \boldsymbol{\psi}_j(t_i)^\top \otimes \boldsymbol{w}_{ij}^\top \Phi_j \right\} \mathrm{vec} B_j + \varepsilon_i \\ &= \boldsymbol{z}_i^\top \boldsymbol{\theta} + \varepsilon_i, \end{aligned} \tag{11}$$

where $\otimes$ denotes a Kronecker product of the matrix that gives

$$A \otimes B = \begin{pmatrix} a_{11}B & \cdots & a_{1m}B \\ \vdots & \ddots & \vdots \\ a_{n1}B & \cdots & a_{nm}B \end{pmatrix}$$

for arbitrary matrices $A = (a_{ij})_{ij}$ $(i = 1, \ldots, n, j = 1, \ldots, m)$ and B, and vec is an operator that aligns a matrix to a vector in the column-wise. Furthermore, $\Phi_j = \int_{\mathcal{T}} \boldsymbol{\phi}_j(s)\boldsymbol{\phi}_j(s)^\top ds$, $\boldsymbol{z}_i = (\boldsymbol{\psi}_1(t_i)^\top \otimes \boldsymbol{w}_{i1}^\top \Phi_1, \ldots, \boldsymbol{\psi}_p(t_i)^\top \otimes \boldsymbol{w}_{ip}^\top \Phi_p)^\top$ is a known vector and $\boldsymbol{\theta} = ((\mathrm{vec}B_1)^\top, \ldots, (\mathrm{vec}B_p)^\top)^\top$ is a vector of unknown parameters. Therefore, the problem of estimating the VCFRM (8) becomes that of estimating $\boldsymbol{\theta}$ in (11) and we can estimate it by traditional estimation strategies. Furthermore, this provides a statistical model for a response vector

$\boldsymbol{y} = (y_1(t_1), \ldots, y_n(t_n))^\top$ as follows:

$$f(\boldsymbol{y}; \boldsymbol{\theta}, \Sigma) = \frac{1}{(2\pi)^{n/2}|\Sigma|^{1/2}} \exp\left\{-\frac{1}{2}(\boldsymbol{y} - Z\boldsymbol{\theta})^\top \Sigma^{-1}(\boldsymbol{y} - Z\boldsymbol{\theta})\right\},$$

where $Z = (\boldsymbol{z}_1, \ldots, \boldsymbol{z}_n)^\top$.

We estimate the unknown parameter $\boldsymbol{\theta}$ by the penalized likelihood method, that is, by maximizing the following penalized likelihood function:

$$\ell_\lambda(\boldsymbol{\theta}, \Sigma) = \ell(\boldsymbol{\theta}, \Sigma) - \frac{n}{2}\boldsymbol{\theta}^\top \Omega_\lambda \boldsymbol{\theta},$$

where $\ell(\boldsymbol{\theta}, \Sigma) = \log f(\boldsymbol{y}; \boldsymbol{\theta}, \Sigma)$ is a log-likelihood function and Ω_λ is a non-negative definite matrix that controls the smoothness of the unknown functions $\beta_j(s,t)$, and it depends on tuning parameters. In our analysis, we denote Ω_λ as $\Omega_\lambda = \text{blockdiag}\{\Omega_1, \ldots, \Omega_p\}$, where blockdiag denotes a block diagonal matrix whose block diagonal elements are matrices in brackets. Furthermore, Ω_j is given by

$$\Omega_j = \lambda_s \Omega_j^{(s)} + \lambda_t \Omega_j^{(t)} \ (j = 1, \ldots, p),$$
$$\Omega_j^{(s)} = \text{blockdiag}\{D_j^{(2)^\top} D_j^{(2)}, \ldots, D_j^{(2)^\top} D_j^{(2)}\}, \quad \Omega_j^{(t)} = \tilde{D}_j^{(2)^\top} \tilde{D}_j^{(2)},$$
$$D_j^{(2)} = \begin{pmatrix} 1 & -2 & 1 & 0 & \cdots & 0 \\ 0 & 1 & -2 & 1 & \cdots & 0 \\ \vdots & \ddots & \ddots & \ddots & \ddots & \vdots \\ 0 & \cdots & 0 & 1 & -2 & 1 \end{pmatrix}, \quad \tilde{D}_j^{(2)} = \begin{pmatrix} -1 & \mathbf{0}_{m^{(1)}-1}^\top & 2 & \mathbf{0}_{m^{(1)}-1}^\top & 1 & \cdots & 0 \\ \vdots & \ddots & \ddots & \ddots & \ddots & \ddots & \vdots \\ 0 & \cdots & -1 & \mathbf{0}_{m^{(1)}-1}^\top & 2 & \mathbf{0}_{m^{(1)}-1}^\top & 1 \end{pmatrix}.$$

Matrices $\Omega_j^{(s)}$ and $\Omega_j^{(t)}$ have rolls for smoothing the coefficient functions $\beta_j(s,t)$ in the s and t directions, respectively, and $\lambda_s, \lambda_t > 0$ are tuning parameters that control the degrees of smoothness for s and t directions respectively.

Maximizing the $\ell_\lambda(\boldsymbol{\theta}, \Sigma)$ with respect to $\boldsymbol{\theta}$, we obtain a maximum penalized likelihood estimator

$$\hat{\boldsymbol{\theta}} = (Z^\top Z + n\Omega_\lambda)^{-1} Z^\top \boldsymbol{y}. \tag{12}$$

The estimator $\hat{\Sigma}$ of the variance-covariance matrix Σ depends on the covariance structure. See Fahrmeir et al. (2013) for details. Using the estimator of $\boldsymbol{\theta}$, denoted by $\hat{\boldsymbol{\theta}} = ((\text{vec}\hat{B}_1)^\top, \ldots, (\text{vec}\hat{B}_p)^\top)^\top$, we obtain estimated coefficient functions, respectively given by

$$\hat{\beta}_j(s,t) = \boldsymbol{\phi}_j(s)^\top \hat{B}_j \boldsymbol{\psi}_j(t).$$

Furthermore, the predicted crop yield for a future time point t_i is given by

$$\hat{y}_i(t_i) = \boldsymbol{z}_i^\top \hat{\boldsymbol{\theta}}.$$

3 Selection of tuning parameters

To obtain the appropriately smooth estimator of $\beta_j(s,t)$ that can easily interpret the relationship between environmental factors and crop yield, we have to select optimal values of tuning parameters λ_s and λ_t. Here we select these values by an information criterion that measures the goodness of the statistical model in the prediction viewpoint. For more details of the information criteria, see Konishi and Kitagawa (2008).

If we have the maximum penalized likelihood estimators of the coefficient parameter $\hat{\boldsymbol{\theta}}$ in (12) and variance-covariance matrix $\hat{\Sigma}$ of the VCFRM, a modified version of Akaike's Information Criterion (mAIC) for evaluating the VCFRM is given by

$$\mathrm{mAIC} = -2\ell(\hat{\boldsymbol{\theta}}, \hat{\Sigma}) + 2\widehat{edf}, \tag{13}$$

where $\widehat{edf}$ is an effective degrees of freedom which is given by $\widehat{edf} = Z(Z^\top Z + n\Omega_\lambda)^{-1}Z^\top$. The $\widehat{edf}$ represents a measure of the complexity of the estimated VCFRM. In this work, we analyze time-series data for the response, which requires us to use CV carefully. Therefore, the information criteria including mAIC are useful tools for evaluating the statistical model. We repeat calculating mAIC (13) for several different values for tuning parameters λ_s and λ_t and then choose them that minimize the value of mAIC.

References

Araki, Y., Konishi, S., Kawano, S., and Matsui, H. (2009). Functional regression modeling via regularized Gaussian basis expansions. *Ann. Inst. Statist. Math.* **61**, 811–833.

Cardot, H. and Sarda, P. (2008). Varying-coefficient functional linear regression models. *Commun. Stat. Theory Methods* **37**, 3186–3203.

Fahrmeir, L., Kneib, T., Lang, S., and Marx, B. (2013). *Regression.* Berlin Heidelberg: Springer.

Green, P. and Silverman, B. (1994). *Nonparametric regression and generalized linear models: a roughness penalty approach.* London: Chapman & Hall/CRC.

Konishi, S. and Kitagawa, G. (2008). *Information criteria and statistical modeling.* New York: Springer.

Wu, Y., Fan, J., and Müller, H. (2010). Varying-coefficient functional linear regression. *Bernoulli* **16**, 730–758.
